# Eye diseases in chronic kidney disease: A nationwide longitudinal case–control study in Sweden

**DOI:** 10.1111/ceo.14464

**Published:** 2024-11-17

**Authors:** Pablo Ballester Dolz, Karin Ålander, Petra Smedberg, Per Vihlborg, Ing‐liss Bryngelsson, Jessica Westerlund, Karim Makdoumi

**Affiliations:** ^1^ Department of Ophthalmology, Faculty of Medicine and Health Orebro University Orebro Sweden; ^2^ School of Medical Sciences Orebro University Orebro Sweden; ^3^ Department of Medical Sciences, Occupational and Environmental Medicine Uppsala University Uppsala Sweden; ^4^ Department of Occupational and Environmental Medicine Uppsala University Hospital Uppsala Sweden; ^5^ Department of Occupational and Environmental Medicine, Faculty of Medicine and Health Orebro University Orebro Sweden

**Keywords:** cataract, chronic kidney disease, eye disease, glaucoma, macular degeneration

## Abstract

**Background:**

Chronic kidney disease (CKD) is a growing health issue that is becoming more prevalent globally, increasing financial cost on healthcare systems. The purpose of this study is to investigate the incidence of eye diseases in patients diagnosed with CKD in Sweden and to evaluate which eye diseases are most likely to develop.

**Methods:**

A longitudinal population‐based retrospective case–control study was conducted including all individuals diagnosed with chronic kidney disease during the time period 2001–2019. A total of 19 455 cases and 38 890 controls were included. For each case, two controls were matched with the same sex, age, and county of residence.

**Results:**

CKD patients had a significantly higher risk of contracting any eye disease compared to individuals without kidney disease HR 1.73 (CI 1.67–1.79), with an elevated risk for all blocks of diagnoses except for glaucoma HR 0.95 (CI 0.85–1.06). However, this condition developed earlier in cases than in controls. Subanalyses showed an increased risk for chronic eye disease patients to develop cataract HR 1.70 (CI 1.63–1.78), other retinal disorders HR 1.86 (CI 1.72–2.02), and retinal vascular occlusions HR 2.08 (CI 1.73–2.51). In general, diagnosis of an eye disease occurred earlier in cases than controls.

**Conclusions:**

The results from this study suggest that CKD patients have an increased risk to develop eye disease. Ocular disease seems to develop considerably earlier in CKD, even without staging the severity of the disease, with particularly high risk of developing retinal diseases and cataracts. Screening for eye disease in CKD should be considered.

## 1 INTRODUCTION

Chronic kidney disease (CKD) is a global public health concern, resulting in a considerable financial burden on healthcare systems.^1^, ^2^, ^3^ CKD is defined as abnormalities of the kidney structure or reduced kidney function with a glomerular filtration rate (GFR) below 60 mL/min per 1.73 m^2^, or the presence of markers of kidney damage for at least 3 months. This definition applies regardless of the underlying cause.^1^

According to recent data, it is estimated that 8% to 16% of the global population suffers from CKD.^2^, ^3^, ^4^ The primary contributing factors to CKD in developed countries are diabetes and hypertension.^1^, ^4^ All stages of CKD are correlated with elevated risks of cardiovascular morbidity, premature mortality, and reduced quality of life.^3^ Previous studies have also shown an association between CKD and several major eye diseases (ED),^5^ such as cataract,^6^, ^7^, ^8^, ^9^ glaucoma,^6^, ^10^, ^11^ age‐related macular degeneration (AMD),^10^, ^12^ and diabetic retinopathy (DR).^5^, ^7^, ^13^, ^14^

It has been proposed that the development of ED in CKD, at least partially, can be explained by similarities in vascular structure, pathophysiological mechanisms, and developmental pathways between the eye and the kidney.^15^ In addition, it has been indicated that ocular involvement in CKD can occur simultaneously (end‐organ damage)^16^, ^17^ or as a consequence of a renal condition leading to secondary eye involvement.^17^

Previous research has indicated that the main mechanisms contributing to ED in CKD are inflammation, atherosclerosis, endothelial dysfunction, and vascular remodelling.^17^ Furthermore, it has been suggested that the eye and the kidney are closely linked because of significant overlap in risk factors of CKD and ED, including old age, diabetes, hypertension, obesity, hyperlipidemia, and smoking.^15^, ^17^, ^18^

The aim of the current study was to analyse the risk for ED in CKD in the Swedish population.

## 2 METHODS

This was a retrospective case–control study, adhering to the tenets of the Declaration of Helsinki, approved by the Swedish Ethical Review Authority (ref: 2023‐02227‐01), investigating the correlation between CKD and ED in Sweden. For diagnosis, the International Classification of Diseases ICD‐10 was used. Data were collected from a previously created database, created from registries of the National Board of Health and Welfare (NBHW) and *Statistics Sweden*. The original database was designed to study the risk for renal and cardiovascular disease after work‐related exposure to particles.

**FIGURE 1:**
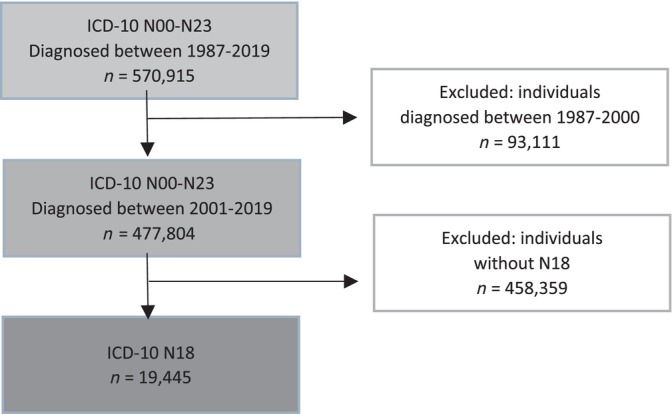
Flowchart of the inclusion‐ and exclusion process for cases.

Cases were identified using the National Inpatient Register and individuals diagnosed with CKD (ICD‐10 N18) during the years 2001–2019 were selected (Figure 1). Every case was matched with two randomly selected controls, that neither had a CKD diagnosis (ICD‐10 N18), nor any other kidney disease diagnosis (ICD‐10 N00‐N23). Cases were matched with individuals with the same sex, age (year of birth), and county of residence at the time of the diagnosis. The National Non‐Primary Outpatient Care Register was used to identify ED. First‐degree relatives to the respective cases were excluded. *Statistics Sweden* conducted the matching process between the registers received from Statistics Sweden and NBHW. Furthermore, the database was matched with the Swedish Emigration Records as well as the National Cause of Death Register, in order to obtain accurate data regarding death and loss of individuals during the study period. All data were anonymized and, therefore, patient information cannot be linked to individuals.

### 2.1 Statistical analysis

The Cox proportional hazards model was used to calculate the risk (HR with 95% CI) of contracting an ED. *p*‐values <0.05 were considered statistically significant. The analyses were subdivided by sex (male/female) and stratified by age at the time of CKD diagnosis into four age groups: Group I (20–30 years), Group II (31–45 years), Group III (46–65 years), and Group IV (66–75 years). Age groups with fewer controls or cases than five individuals were not presented in the results.

Individual analyses (of HR) were performed for all diagnosis blocks in Chapter VII, ICD‐10. Moreover, analyses were done on category‐level for senile cataract and other cataracts (ICD‐10 H25‐H26), retinal vascular occlusions (ICD‐10 H34), other retinal disorders (ICD‐10 H35), retinal disorders in diseases classified elsewhere (ICD‐10 H36), which includes DR (H36.0), and glaucoma (ICD‐10 H40). For the analysis of H36, only cases and controls diagnosed with diabetes (ICD‐10 E10‐E14) before the time of CKD diagnosis were included.

## 3 RESULTS

A total of 58 335 individuals were included in the study (19 445 cases and 38 890 controls), 67% were men and 32% were women. The mean age at the time of CKD diagnosis was 60 years (SD 12.89), median 63 years (range 20–75 years). During the study period, 10 212 cases and 6747 controls were deceased. On average, the cases were 6 years younger than the controls at the time of death (Table 1).

**TABLE 1:** Study population. Cases have chronic kidney disease, ICD‐10 N18.

	Sex	Controls cases	*N*	Mean	Median	Minimum	Maximum	SD
Age when diagnosed	Men	Controls	27 730	61	64	20	75	12.41
		Cases	13 865	61	64	20	75	12.41
	Women	Controls	11 160	58	62	20	75	13.84
		Cases	5580	58	62	20	75	13.84
	Total	Controls	38 890	60	63	20	75	12.89
		Cases	19 445	60	63	20	75	12.89
Age at death	Men	Controls	5172	74	75	23	93	8.65
		Cases	7373	68	70	20	90	8.88
	Women	Controls	1575	75	77	21	93	9.40
		Cases	2839	68	70	21	90	9.84
	Total	Controls	6747	74	75	21	93	8.9
		Cases	10 212	68	70	20	90	9.16

The risk for the development of any ED was significantly higher in cases than controls with a HR of 1.73 (CI 1.67–1.79). When stratified by age, the increased risk remained significant in all age groups. The HR for Group I were 3.72 (CI 2.92–4.74), for Group II 3.79 (CI 3.32–4.33), for Group III 2.23 (CI 2.12–2.35), and for Group IV 1.46 (CI 1.39–1.54) (Table 2).

**TABLE 2:** Hazard ratio (cox regression) for cases (with chronic kidney disease, ICD‐10 N18) and controls for ICD‐10 H00‐H59 (Diseases of the eye and adnexa).

			Total	Eye diseases	HR	95% CI
	Total	Cases	19 445	4994	1.73	1.67–1.79
		Controls	38 890	10 213	1	
	Men	Cases	13 865	3350	1.77	1.70–1.85
		Controls	27 730	6694	1	
	Women	Cases	5580	1644	1.64	1.55–1.74
		Controls	11 160	3519	1	
Age	20–30	Cases	822	169	3.72	2.92–4.74
		Controls	1644	107	1	
	31–45	Cases	2067	530	3.79	3.32–4.33
		Controls	4134	392	1	
	46–65	Cases	8316	2282	2.23	2.12–2.35
		Controls	16 632	3695	1	
	66–75	Cases	8240	2013	1.46	1.39–1.54
		Controls	16 480	6019	1	

**FIGURE 2:**
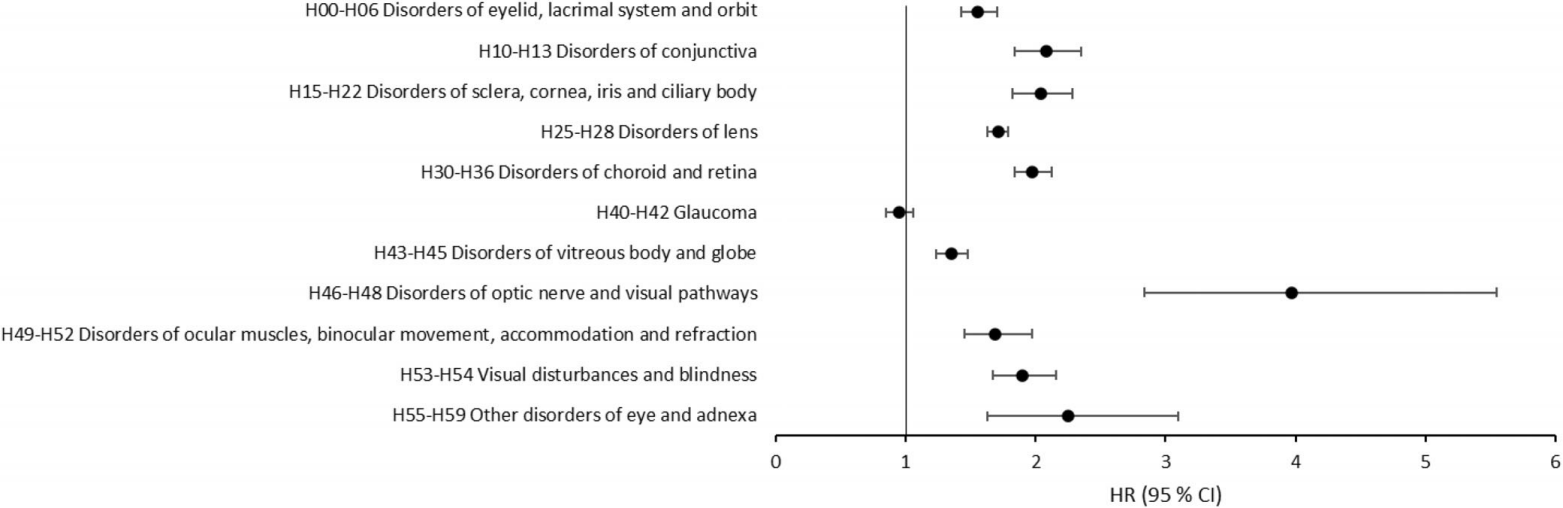
Graph depicting the HR (95% CI) of all the sections of the eye diagnosis chapter in the ICD‐10 for contracting an eye disease after a previous diagnosis of chronic kidney disease (ICD‐10 N18) during the years 2001–2019 in the Swedish population. X = 1 represents the control groups. HR >1 reflects increased risk.

All the ED (ICD‐10 Chapter VII, H00‐H59) were subdivided into the ICD‐10 diagnosis blocks and presented accordingly. A significantly increased risk of developing an ED in cases compared to controls was observed in all sections of ED except for glaucoma (H40‐H42) (Figure 2).

The elevated risk remained for all age groups in the following ICD‐10 blocks: disorders of eyelid, lacrimal system and orbit (H00‐H06), disorders of conjunctiva (H10‐H13), disorders of sclera, cornea, iris, and ciliary body (H15‐H22), disorders of choroid and retina (H30‐H36), and visual disturbances and blindness (H53‐H54) (Table 3).

**TABLE 3:** Hazard ratio (cox regression) for cases (with chronic kidney disease, ICD‐10 N18) and controls for each section of the eye diagnosis chapter in ICD‐10.

ICD‐10 diagnosis	Age		Eye diseases	HR	95% CI
H00‐H06 (disorders of eyelid, lacrimal system, and orbit)	20–30	Cases	30	2.46	1.46–4.16
		Controls	26	1	
	31–45	Cases	95	2.46	1.85–3.27
		Controls	94	1	
	46–65	Cases	376	1.95	1.71–2.22
		Controls	636	1	
	66–75	Cases	253	1.28	1.11–1.47
		Controls	916	1	
H10‐H13 (disorders of conjunctiva)	20–30	Cases	22	2.02	1.13–3.63
		Controls	23	1	
	31–45	Cases	72	2.72	1.94–3.82
		Controls	63	1	
	46–65	Cases	212	2.41	2.01–2.89
		Controls	277	1	
	66–75	Cases	104	1.57	1.25–1.97
		Controls	300	1	
H15‐H22 (disorders of sclera, cornea, iris, and ciliary body)	20–30	Cases	30	2.49	1.47–4.21
		Controls	26	1	
	31–45	Cases	112	3.25	2.45–4.33
		Controls	82	1	
	46–65	Cases	240	2.09	1.77–2.47
		Controls	355	1	
	66–75	Cases	124	1.50	1.22–1.80
		Controls	359	1	
H25‐H28 (disorders of lens)	20–30^a^				
	31–45	Cases	169	15.91	10.65–23.75
		Controls	28	1	
	46–65	Cases	1289	2.81	2.61–3.02
		Controls	1710	1	
	66–75	Cases	4273	1.60	1.51–1.71
		Controls	1405	1	
H30‐H36 (disorders of choroid and retina)	20–30	Cases	61	21.99	9.51–50.89
		Controls	6	1	
	31–45	Cases	150	12.35	8.29–18.39
		Controls	29	1	
	46–65	Cases	546	2.67	2.39–2.99
		Controls	681	1	
	66–75	Cases	466	1.55	1.40–1.73
		Controls	1441	1	
H40‐H42 (glaucoma)	20–30^a^				
	31–45	Cases	33	1.98	1.25–3.13
		Controls	41	1	
	46–65	Cases	192	1.26	1.06–1.49
		Controls	505	1	
	66–75	Cases	940	0.88	0.75–1.03
		Controls	189	1	
H43‐H45 (disorders of vitreous body and globe)	20–30^a^				
	31–45	Cases	70	3.71	2.55–5.38
		Controls	46	1	
	46–65	Cases	383	1.47	1.30–1.66
		Controls	820	1	
	66–75	Cases	158	0.97	0.81–1.15
		Controls	643	1	
H46‐H48 (disorders of optic nerve and visual pathways)	20–30^a^				
	31–45^a^				
	46–65	Cases	38	4.75	2.81–8.02
		Controls	23	1	
	66–75	Cases	18	2.21	1.24–3.93
		Controls	38	1	
H49‐H52 (disorders of ocular muscles, binocular movement, accommodation, and refraction)	20–30	Cases	24	2.20	1.24–3.90
		Controls	23	1	
	31–45	Cases	54	1.81	1.27–2.59
		Controls	70	1	
	46–65	Cases	126	1.71	1.37–2.13
		Controls	224	1	
	66–75	Cases	53	1.28	0.94–1.75
		Controls	182	1	
H53‐H54 (visual disturbances and blindness)	20–30	Cases	17	2.41	1.20–4.82
		Controls	15	1	
	31–45	Cases	56	3.37	2.24–5.05
		Controls	40	1	
	46–65	Cases	158	2.05	1.68–2.51
		Controls	253	1	
	66–75	Cases	131	1.72	1.40–2.12
		Controls	349	1	
H55‐H59 (other disorders of eye and adnexa)	20–30^a^				
	31–45	Cases	15	3.80	1.66–8.70
		Controls	9	1	
	46–65	Cases	33	3.17	1.94–5.16
		Controls	33	1	
	66–75	Cases	13	1.19	0.64–2.21
		Controls	50	1	

^a^ Unsubstantial amount of affected individuals to present any result.

For disorders of ocular muscles, binocular movement, accommodation, and refraction (H49‐H52) there was no difference in risk for Group IV. Regarding disorders of the vitreous body and globe (H43‐H45) and other disorders of the eye and adnexa (H55‐H59), there was a significantly increased of risk in age Groups II and III, but not in Group IV. The number of cases was insufficient for analysis in Group I. In disorders of the lens (H25‐H28), the risk was significantly higher in cases than controls (1.71 CI 1.63–1.79). This was observed in all age groups except for Group I, where the sample size was too small. There were also insufficient numbers of individuals in the age Groups I and II in the diagnosis block comprising disorders of the optic nerve and visual pathways (H46‐H48). However, there was an increased risk the age Groups III and IV. The results for H40‐H42 (glaucoma) were identical to H40 (glaucoma) (Table 3).

### 3.1 Correlation between CKD and major eye diseases

#### 3.1.1 Senile cataract and other cataract (H25‐H26)

Hazard ratio for the diagnosis of cataract was higher in cases compared to controls (HR 1.70, CI 1.63–1.78). However, since the database did not include information on cataract surgery, no analysis on the time point of cataract surgery was possible. Segmentation by age demonstrated an increased risk in all age groups except Group I (insufficient number of individuals). The HR was 15.55 (CI 10.41–23.23) for Group II, 2.81 (CI 2.61–3.02) for Group III and 1.60 (CI 95% 1.50–1.70) for Group IV.

#### 3.1.2 Retinal vascular occlusions, including retinal vein occlusion (H34)

The data analysis regarding the risk of contract retinal vascular occlusions revealed a significantly increased risk for cases compared to controls (HR 2.08, CI 1.73–2.51). Age stratification showed a significant risk in age groups III (HR 2.85, CI 2.10–3.86) and IV (HR 1.89, CI 1.45–2.46). The number of individuals was too scarce for statistical analysis in age groups I and II.

#### 3.1.3 Other retinal disorders, including AMD (H35)

The risk of developing other retinal disorders, which include AMD, was significantly higher in cases than controls (HR 1.86, CI 1.72–2.02), with the highest risk observed in age Group I (HR 20.87, CI 9.00–48.37). The risk decreased with age, with HRs of 9.87 (95% CI 6.26–15.55) for Group II, 2.87 (CI 2.50–3.29) for Group III, and 1.47 (1.30–1.66) for Group IV.

#### 3.1.4 Retinal disorders classified elsewhere, including diabetic retinopathy (H36)

There was no significant difference for cases with diabetes prior to CKD diagnosis to contract retinopathy compared to diabetic controls without CKD (HR 0.99, CI 0.51–1.93). These analyses were, due to an insufficient number of individuals, not possible to stratify by age.

#### 3.1.5 Glaucoma (H40)

No significant difference was detected between cases and controls regarding the risk of developing glaucoma (HR 0.95 and 95% CI 0.85–1.06). However, there was a higher risk both age Groups II (HR 1.98, CI 1.25–3.13) and III (HR 1.26, CI 1.06–1.49). No difference was observed in Group IV and there was an inadequate number of study subjects in Group I.

## 4 DISCUSSION

Our study supports the findings from previous population‐based research, which have demonstrated an association between CKD and ED.^5^, ^7^ However, this is, to our knowledge, the first study to analyse the correlation between CKD and all respective groups of ED, as defined by ICD‐10, chapter VII.

We found that patients with CKD had higher risk of developing any ED (HR 1.73). In major eye disease, results showed higher risk of developing cataract (HR 1.70), macular degeneration, or other retinal disorders (HR 1.84) and retinal vascular occlusions, including retinal vein occlusion (HR 2.08). These findings are consistent with previous studies that have showed significant correlation between CKD and eye diseases,^5^, ^7^ cataract,^6^, ^7^, ^8^, ^9^ AMD,^10^, ^12^ and retinal vascular disorders.^19^, ^20^, ^21^ A national cross‐sectional study in United States reported that the prevalence of major eye diseases was ~2–7‐fold higher in participants with CKD than in those without the condition (*p* < 0.05).^5^ In addition, significant associations were found between CKD and any ocular diseases, including cataract, retinopathy, and DR, in a population‐based study from Singapore. The age and sex‐adjusted OR for any ocular disease was 1.54 (95% CI 1.24–1.91, *p* < 0.001).^7^ A case–control study in Taiwan found that patients with CKD had higher risk of developing cataract with an OR of 1.24 (95% CI 1.18–1.31, *p* < 0.001).^6^ An age and gender‐matched study of Stages 3 to 5 CKD of 150 hospital patients from Australia described that renal impairment was an independent risk factor for non‐exudative macular degeneration, OR 1.79 (95% CI 1.00–3.20, *p* < 0.05).^22^ In another Australian investigation, the population‐based prospective Blue Mountains study, individuals with moderate CKD had three times higher risk to develop early AMD than those with no CKD OR 3.2 (95% CI 1.8–5.7, *p* < 0.0001).^23^ Furthermore, two matched cohort studies from South Korea^19^ and Taiwan^21^ including 988 and 904 patients with end‐stage renal disease (ESRD), respectively, both reported an association between the condition and retinal vein occlusion HR 2.122 (95% CI 1.396–3.226, *p* = 0.0004) and HR 3.05 (95% CI 2.64–3.51).

However, our analyses did not find a significantly higher risk of developing glaucoma in patients with CKD. Previous studies have reported conflicting results regarding whether CKD increases the risk of glaucoma. Two cross‐sectional studies, both from Singapore,^7^, ^24^ reported no elevated risk of developing glaucoma in CKD patients. In contrast, a nationwide Korean cohort study^11^ described that patients with CKD had higher risk to contract glaucoma with an overall HR 1.63 (95% CI 1.34–1.98). Possible explanations for these conflicting results might be differences in study methodologies, including number of participants and ethnicities of populations. It may be a possibility that CKD patients have an increased risk of contracting glaucoma earlier at an earlier age than individuals with normal kidney function. This would be consistent with our results, as we observed an augmented risk between the ages of 31 to 65 years (HR 1.98, for age Group II and HR 1.26 in the age Group III), but not in the highest age group (66–75 years). Further investigations are needed to address the varying associations by ethnicity and importance of age, given that previous studies were conducted in Asia and ethnicity has been established as a significant effect moderator in glaucoma.^25^

In the present analyses, we found no difference in risk for people with diabetes and CKD to develop DR compared to diabetic controls without kidney disease. This result differs from previous population‐based studies.^5^, ^7^, ^13^, ^14^ A possible explanation as to why our result differs from previous studies could be that it is limited by the small number of patients in the subanalysis for DR. It is, however, important to emphasise that we focused specifically on the risk for the development of DR in cases versus controls with diabetes mellitus. This signifies that we only evaluated the risk for individuals with CKD in the diabetic population, not the risk for DR secondary to CKD compared to non‐diabetic controls.

By stratifying our results by age, our data showed that in general, there was an increased risk for the development of ED in the younger age groups. This was especially apparent for disorders of the lens, including cataract (HR 15.55 for age Group II), and other retinal disorders, including macular degeneration (HR 20.87 in age Group I). Our interpretation of the findings is that CKD patients seem prone to developing these conditions at an earlier age compared to controls without kidney disease, who likely develop such age‐related diseases later in life.

Some strengths in this study, are the longitudinal study design, the size of the study population, and the matching of cases and controls. Furthermore, we subdivided the study groups into age categories which allowed us to evaluate differences with regards to age at the development of ED. We consider this aspect relevant as it highlights that patients with CKD seem to develop ED at a lower age than the general population. Our study is also unique because it includes the entire Swedish population, which is relatively diverse in terms of ethnicity, and we included all stages of CKD. Since we followed individuals after the initial diagnosis of CKD, it seems reasonable to assume that a relatively large proportion of cases were not suffering from a more severe CKD or ESRD. Still, the risk for ED was relatively similar to what has been reported in previous studies.

However, the results of our study should be considered in the context of some limitations. All diagnoses, including CKD and conditions classified as ED, were collected from the National Patient Register, made in clinical practice and consequently, there is a degree of uncertainty regarding the specificity of conditions. Nonetheless, diagnoses were collected from data recorded in the national non‐primary outpatient visit register, which is considered more accurate than diagnoses based on questionnaires.^26^ Moreover, a diagnosis is not always representative of the clinical course of a disease and might be revised after it is made. Most patients with early stages of CKD are asymptomatic, which in this study may lead to a certain risk of cases classified as controls. However, this would imply that, the risk may be somewhat greater than our reported results. In contrast, since the National Inpatient Register was used to identify cases, there is a risk that some individuals with diagnosed CKD were not included in the study and that the timing of diagnosis may have been delayed until these individuals were admitted into a hospital clinic. Nevertheless, The Swedish National Patient Register is a reliable tool for epidemiological studies, enabling the possibility of retrieving morbidity data for the entire patient population.^26^ Despite the inherently low accuracy, relying on ICD‐10 diagnoses and the imprecise definition of CKD a significant association between CKD and ED was still established. In addition, our data did not take different stages of CKD into consideration and to be able to understand how CKD progression affects the eye, studies stratification of the CKD stages is needed. Lastly, since our study did not include an analysis of confounders, except for age, sex, and county, it was not possible to conduct a detailed investigation of the influence of other factors influencing the association between CKD and ED. However, the aim of the study was to investigate the correlation between CKD and ED, regardless of the causes of eye disorders in patients with CKD. Due to patients with CKD has a higher risk of developing any ED, which plausibly has an impact on quality of life, it may be relevant to consider screening for ED in this population, in particular regarding cataracts and retinal diseases.

In conclusion, CKD patients were generally more affected by ED than individuals without kidney disease. The risk was higher in the younger age groups, especially for disorders of the lens, including cataracts as well as disorders of the choroid and retina, comprising macular degeneration. In addition, there was a significantly higher risk of developing glaucoma in patients with CKD in the ages 31–65 years old. Further studies are required to reveal the underlying risk factors and pathophysiological mechanisms contributing to specific ED in CKD patients. Given the increased risk for ED in CKD we suggest that screening for ocular disease in CKD should be considered.

## FUNDING INFORMATION

None.

## CONFLICT OF INTEREST STATEMENT

The authors declare no conflicts of interest.

## Data Availability

The data that support the findings of this study are available from the corresponding author upon reasonable request.
